# Circular Shoulder Defect: Use of L-Shaped Stacked LD Myocutaneous Flap

**DOI:** 10.1055/s-0044-1800781

**Published:** 2024-11-28

**Authors:** Rajesh Yellinedi, Mukunda Reddy Damalachervu, Rambabu Nuvvula, Subramanyeshwar Rao Thammineedi, Srinath Gupta

**Affiliations:** 1 Department of Plastic and Reconstructive Surgery, Basavatarakam Indo American Cancer Hospital and Research Institute, Hyderabad, Telangana, India; 2 Department of Surgical Oncology, Basavatarakam Indo American Cancer Hospital and Research Institute, Hyderabad, Telangana, India; 3 Department of Surgical Oncology, Basavatarakam Indo American Cancer Hospital and Research Institute, Hyderabad, Telangana, India

Wide excision of tumors on the shoulder often lead to large circular skin defects exposing varying amount of shoulder girdle. The pedicled latissimus dorsi (LD) myocutaneous flap is very useful to cover these defects 1 and also provide laxity to maintain full range of shoulder motion. However only a single (transverse, vertical, or oblique oriented)-axis skin paddle may not be enough to cover the entire circular defect on the shoulder. 1 2 However, if a larger skin paddle is harvested, the donor area cannot be closed primarily.

We present our method of using an L (a combination of vertical and horizontal paddles)-shaped skin paddle of the LD myocutaneous flap to reconstruct these defects while maintaining donor site primary closure. The defect's global area is split and calculated as two limbs of L. The horizontal portion is planned in the lower half of the muscle and the vertical portion in the lateral area connected by an L pattern. The base of the L is fashioned at the flank region. Only the amount of width amenable for primary closure (about 5–6 cm in each axis by pinch) is taken. Length may vary depending on body habitus and requirement. The rest of the flap harvest is done by islanding on the thoracodorsal vessels and intact insertion of the tendon to prevent shearing. The flap is tunneled across the remaining skin bridge to reach the defect. The two free ends of the L are then stacked side-by-side to create one large skin paddle enough to cover the circular defect and also achieve donor site primary closure. For example, in a 10-cm diameter defect, harvesting a single 10 cm wide LD skin paddle can make donor site closure not possible. By using two 5-cm wide paddles folded together closure is possible at all areas.

**Fig. 1:**
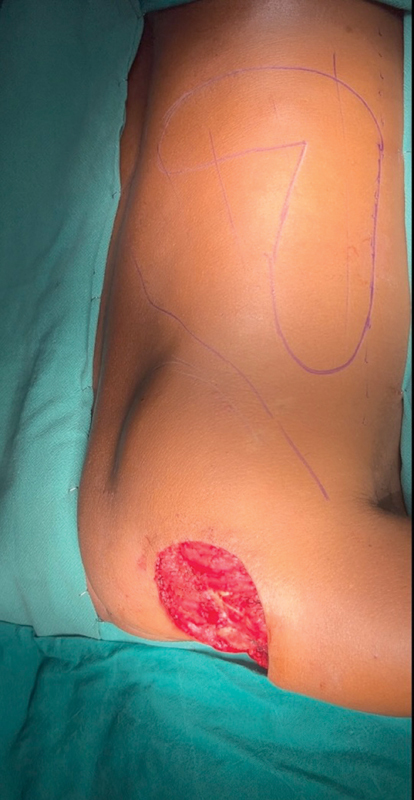
Wide excision defect on shoulder with L skin paddle design.

Between 2019 and 2023 we had reconstructed three such cases in such manner ( Figs. 1 2 3 4 ). Age group was between 40 and 45 years, manual workers with tough skin. All the flaps healed well. Adjuvant radiation therapy was started 1 month after surgery and tolerated well without any wound breakdown. One-year follow-up had ( Video 1 , demonstrating planning procedure and follow-up) complete passive range of motion across the shoulder joint without any limitation from skin shortage or contracted skin grafts.

**Fig. 2:**
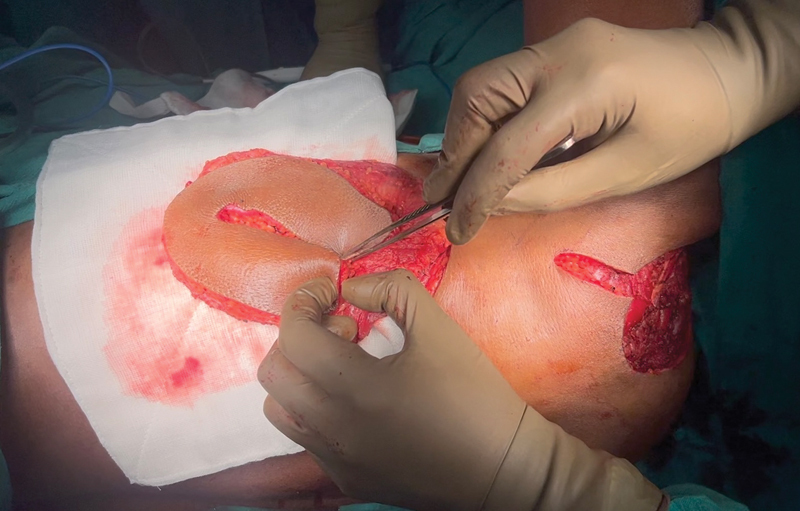
Harvested flap with folded skin paddle.

**Fig. 3:**
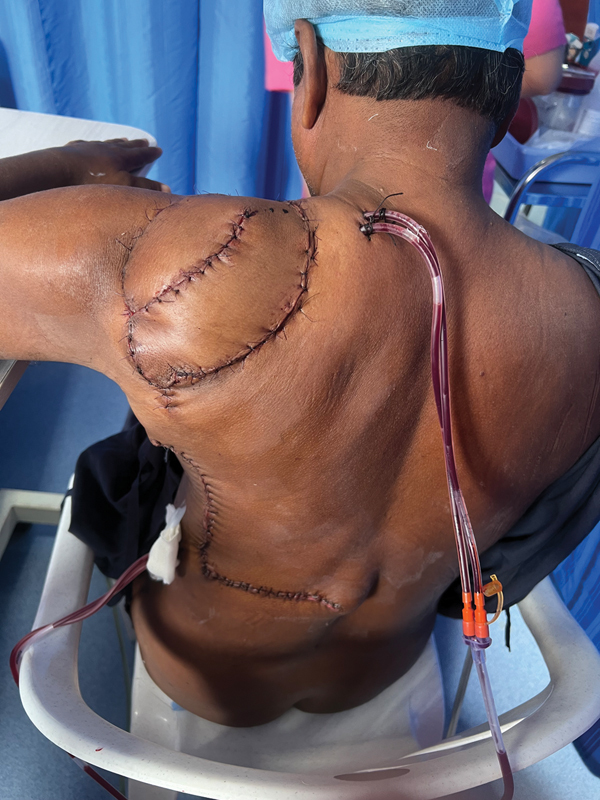
Postop result with stacked L-shaped latissimus dorsi (LD) myocutaneous flap and donor site primary closure.

**Fig. 4:**
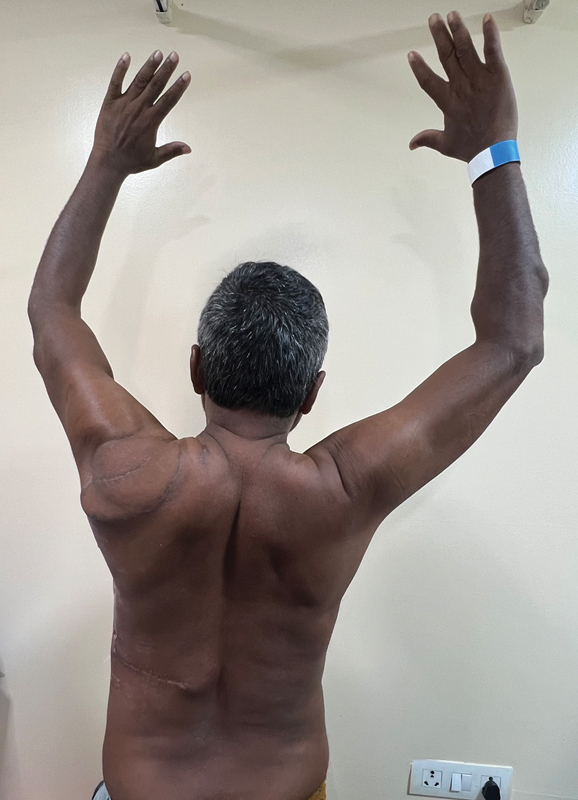
One-year follow-up.

**Video 1** Video demonstrating markings, fashioning, and final inset.


Zhang et al described a bilobed and trilobed design of skin paddle stacked to increase surface area of skin paddle. However, they were used as free flaps to cover predominantly scalp defects. 3 Schaverien et al used a computed tomography perfusion study to show that the vertical and lower transverse skin paddles were perfused by the descending branch of the thoracodorsal artery making this design robust. 4 Thoracodorsal artery perforator flaps 5 based on multiple perforators can be used in a similar fashion, but our design without any perforator identification makes it simple. Thus, the L-shaped stacked skin paddle provides a simple solution for coverage of circular shoulder defects, achieve good shoulder contour, and donor site primary closure.
